# Association between chronic pancreatitis and urolithiasis: A population-based cohort study

**DOI:** 10.1371/journal.pone.0194019

**Published:** 2018-03-09

**Authors:** Chien-Hua Chen, Cheng-Li Lin, Long-Bin Jeng

**Affiliations:** 1 Digestive Disease Center, Show-Chwan Memorial Hospital, Changhua, Taiwan; 2 Digestive Disease Center, Changbing Show-Chwan Memorial Hospital, Lukang Township, Changhua County, Taiwan; 3 Department of Food Science and Technology, Hungkuang University, Taichung, Taiwan; 4 Chung Chou University of Science and Technology, Yuanlin Township, Changhua County, Taiwan; 5 Management Office for Health Data, China Medical University Hospital, Taichung, Taiwan; 6 College of Medicine, China Medical University, Taichung, Taiwan; 7 Graduate Institute of Clinical Medical Science, School of Medicine, College of Medicine, China Medical University, Taichung, Taiwan; 8 Department of Surgery, Organ Transplantation Center, China Medical University Hospital, Taichung, Taiwan; University of Cincinnati College of Medicine, UNITED STATES

## Abstract

**Purpose:**

Chronic pancreatitis (CP) can cause fat or bile acid malabsorption due to exocrine insufficiency. Fat or bile acid malabsorption has been reported to increase the risk of urolithiasis through increased intestinal oxalate absorption. However, no studies have reported an association between CP and urolithiasis.

**Methods:**

We identified 15,848 patients (age: ≥20 years) diagnosed as having CP between 2000 and 2010 from the National Health Insurance Research Database as the study cohort. Beneficiaries without a history of CP were randomly selected and propensity-matched with the study cohort in a 1:4 ratio according to age; sex; comorbidities of hyperlipidemia, diabetes, obesity, hypertension, chronic obstructive pulmonary disease, alcohol-related illness, stroke, and coronary artery disease; and the index date. The prevalence of inflammatory bowel disease (0.44%), hyperparathyroidism (0.10%), or end stage renal disease (1.55%) in CP patients was low, but these comorbidities were also considered in the analysis. All patients were followed until the end of 2011 or withdrawal from the National Health Insurance program to determine the incidence of urolithiasis.

**Results:**

The cumulative incidence of urolithiasis was higher in the CP cohort than that in the non-CP cohort (log-rank test, P < 0.001) with a 1.89-fold risk of urolithiasis (95% confidence interval [CI] = 1.74–2.06). The prevalence of CP was higher in men (81.9%) and in patients younger than 49 years (63.5%; mean age: 48.5 ± 15.3 years). CP was associated with the development of urolithiasis in each age group (≤49 years: aHR = 2.00, 95% CI = 1.81–2.22; 50–64 years: aHR = 1.71, 95% CI = 1.40–2.09; ≥65 years: aHR = 1.54, 95% CI = 1.20–1.98) and each sex (women: aHR = 2.10, 95% CI = 1.67–2.66; men; aHR = 1.86, 95% CI = 1.70–2.04). Among the patients without comorbidities, the rate of urolithiasis increased from 2.93/1,000 person-years in non-CP patients to 8.28/1,000 person-years in CP patients. Among the patients with comorbidities, the rate of urolithiasis increased from 6.12/1,000 person-years in non-CP patients to 10.9/1,000 person-years in CP patients. The contribution of CP to the relative risk of urolithiasis was greater in patients without comorbidities (without comorbidities: aHR = 2.81, 95% CI = 2.30–3.44) than in those with comorbidities (aHR = 1.76, 95% CI = 1.61–1.94).

**Conclusion:**

CP is associated with urolithiasis in this population-based cohort study. The contribution of CP to the relative risk of urolithiasis was even greater in patients with a lower risk of urolithiasis, such as those without other comorbidities. Our findings warrant a survey and education on urolithiasis for patients with CP.

## Introduction

Urolithiasis involves stone formation in the urinary system, namely the kidney, ureter, urinary bladder, and urethra. The prevalence means any stone during a period of life and the lifetime prevalence of urolithiasis would increase with incremental age [1]. The incidence of urolithiasis varies with age and it is rare before age 20; nevertheless, urolithiasis would peak between age 40–60 and then decrease after age 65 [2]. The annual incidence of urolithiasis is reported to range between 0.5% and 1.5% in Western countries, but the epidemiological studies of incidence in Taiwan is limited [3, 4]. In Asian countries, the reported annual incidence of urolithiasis is 134.0 per 100,000 population in Japan and the 11-year cumulative incidence is 5.71% with the incidence higher in men (7.07% vs. 4.34%) in Korea [5, 6]. Urolithiasis is one of the most common diseases accounting for hospital visits in the urology department, and approximately 25% of patients with urolithiasis experience recurrent renal colic after the first episode [7]. In urolithiasis, the stones can migrate downward through the urinary tract, and this can lead to renal colic, urinary tract infection, renal function impairment, and even end stage renal failure. Urolithiasis has markedly affected quality of life and caused socioeconomic burden; therefore, the identification and retrieval of urolithiasis are important to mitigate public health concerns [7].

Chronic pancreatitis (CP) is characterized by a chronic progressive inflammation of the pancreas that results in irreversible pancreas damage, which can cause endocrine or exocrine insufficiency [8]. The diagnosis of CP is mainly based on clinical and imaging criteria, because pathological findings are typically unavailable and the pancreatic function test is quite cumbersome [9–11]. Most published diagnostic criteria are modified according to the initial Cambridge classification system such as pancreatic calcifications, pancreatic stones, and pancreatic duct stenosis or dilatation in endoscopic retrograde cholangiopancreatography, endoscopic ultrasound, computed tomography, or magnetic resonance imaging [10, 11]. The triad of CP include abdominal pain, exocrine pancreatic insufficiency, and diabetes. However, abdominal pain is the common symptom and it is present in approximately 85% of patients with CP [11].

The incidence of CP has slightly increased worldwide with the greater availability of diagnostic imaging techniques, awareness of CP, and alcohol consumption [12]. CP is relatively prevalent in Asia, the reported prevalence is 125/100,000 in India and the prevalence in China has increased from 3.08/100,000 in 1996 to 13.52/100,000 in 2003 [13, 14]. Alcohol consumption remains the most common cause (70%–80%) of CP worldwide, although the cause of CP is deemed multifactorial and genetic factors also play a very important role in the pathogenesis of CP [15, 16]. The etiology of CP varies in different Asia-Pacific countries as idiopathic pancreatitis is the most common cause reported from China and India, but alcohol accounts for approximately 95% in Australia and 54% in Japan, respectively [13, 14, 17]. Fat malabsorption is the most pronounced presentation of exocrine insufficiency of CP because of the previous decrease in lipase secretion, greater susceptibility of lipase to the acidic intestinal environment and proteolytic destruction, acidic denaturation of the bile acids, and marked inhibition of bile acid secretion in CP [18]. Furthermore, studies have reported that approximately 80%–90% of patients with CP exhibited some degree (50%–80%) of exocrine insufficiency, although overt steatorrhea, weight loss, and abdominal discomfort would only develop in advanced stages of CP [19, 20].

Stones are categorized according to their mineral components, and calcium oxalate stones are the most common in urolithiasis [21]. Fat or bile acid malabsorption was reported to increase the risk of urolithiasis through increased intestinal oxalate absorption [22]. Not only an impaired function of the small bowel such as small bowel resection and bypass surgery but also a defective secretion of digestive enzymes such as CP and blind loop syndrome can cause fat or bile acid malabsorption. Unabsorbed bile acids and fatty acids will bind with the intestinal calcium to produce saponification, which will limit the binding between free calcium and oxalate within the intestinal lumens. The consequent increase in intestinal oxalate absorption will result in hyperoxaluria to increase the risk of oxalate urolithiasis [23]. Furthermore, it has been reported that bile acids may directly increase the colonic permeability of oxalate into the blood vessels [24].

In addition to CP being closely related to fat or bile acid malabsorption through exocrine insufficiency, alcohol consumption is the most common cause of CP and is related to the development of uric acid stones. However, no studies have reported an association between CP and urolithiasis. In this study, we hypothesized that CP might be associated with an increased risk of urolithiasis. We conducted a nationwide population-based cohort study by analyzing data from Taiwan’s National Health Insurance Research Database (NHIRD) to assess the aforementioned association.

## Methods

### Data source

This nationwide cohort study was conducted using the NHIRD of the National Health Insurance (NHI) program in Taiwan [25]. This program was established on March 1, 1995, and it covers more than 99.5% of the 23.74 million residents of Taiwan. The NHIRD contains comprehensive health care data on outpatient and inpatient claims from the NHI program, whose enrollment record is stored [26, 27]. The NHIRD (http://w3.nhri.org.tw/nhird//date_01.html) is maintained by a government-established nonprofit organization, the National Health Research Institutes (http://nhird.nhri.org.tw/). All the data of the NHIRD are encrypted and provided to researchers after approval of formal applications. For this retrospective cohort study, we used a subset of the NHIRD containing health care data including files of inpatients claims, and Registry of Beneficiaries. The 2001 International Classification of Diseases, Ninth revision, Clinical Modification (ICD-9-CM) was used for disease coding.

### Ethics statement

The NHIRD encrypts patient personal information to protect privacy and provides researchers with anonymous identification numbers associated with relevant claims information, including sex, date of birth, medical services received, and prescriptions. Therefore, patient consent is not required to access the NHIRD. This study was approved to fulfill the condition for exemption by the Institutional Review Board (IRB) of China Medical University (CMUH104-REC2-115-CR2). The IRB also specifically waived the consent requirement.

### Patients

This study recruited patients aged ≥20 years who were newly diagnosed as having CP (ICD-9-CM: 577.1) based on the inpatients claims between January 1, 2000, and December 31, 2010. The date of CP diagnosis was defined as the index date. The control cohort comprised patients without a history of pancreatitis and was randomly selected from the NHIRD through 4:1 matching with the study cohort. Patients in the non-CP (ICD-9-CM: 577) cohort were frequency-matched according to age (in 5-y intervals); sex; comorbidities of obesity, hyperlipidemia, diabetes, hypertension, chronic obstructive pulmonary disease (COPD), alcohol-related illness, stroke, and coronary artery disease (CAD); and the index year. The prevalence of inflammatory bowel disease (0.44%), hyperparathyroidism (0.10%), or end stage renal disease (1.55%) in CP patients was low, but these comorbidities were also considered in the analysis. Both the CP and non-CP cohorts excluded patients with a history of urolithiasis (ICD-9-CM: 592 and 594) and those with incomplete information on age or sex during enrollment. Each subject was monitored from the index date until a new diagnosis of urolithiasis or until the subject was censored because of death, withdrawal from insurance, or the end of follow-up on December 31, 2011. The major reasons for withdrawal from the NIH program included emigration from Taiwan and death. The coding for identified cause-specific and non-cause-specific deaths was included in the analysis; however, it was censored if the causes could not be identified. The control patients were randomly selected according to the month and day with the same index year as the matched patients.

### Covariates of interest

The NHRI classified all city districts and townships of Taiwan into 7 urbanization levels according to population density (people/km^2^), proportion of residents with higher education, elderly and agricultural population, and the number of physicians per 100,000 people in each area [28]. Level 1 stood for areas with a higher population density and socioeconomic status, whereas level 7 stood for the lowest. Because few people lived in the rural areas between levels 4–7, we grouped these areas into the level 4 group. White-collar workers were employees with indoor works such as public institutional workers, educators, or administrative personnel in business and industries. Blue-collar workers were characterized by longer hours of outdoor works such as fishermen, farmers, or industrial laborers. Those subjects with other occupations included primarily retired, unemployed, and low-income populations [29].

### Statistical analysis

The chi-squared test was used for the comparison and examination of the distributions of age, sex, and comorbidities between the CP and non-CP cohorts. The Student t test was used for comparing the mean ages (standard deviations [SDs]) and follow-up periods (SDs) between the two cohorts. In addition, the Kaplan–Meier method was used to compare the cumulative incidence of urolithiasis events and survival between the study and control cohorts, and the differences were examined using the log-rank test. The incidence density rates of urolithiasis were estimated by dividing the number of urolithiasis events by the number of person-years for each risk factor and subsequently stratified by age, sex, and comorbidities. The risk of CP-associated urolithiasis was assessed using univariable and multivariable Cox proportional hazards regression models. The corresponding hazard ratios (HRs) and 95% confidence intervals (CIs) were estimated using a Cox model adjusted for age, sex, occupation, urbanization level, hyperlipidemia, diabetes, hypertension, COPD, alcohol-related illness, stroke, and CAD. SAS Version 9.4 (SAS Institute, Cary, NC, USA) was used for data analyses, and a two-tailed P value of <0.05 was considered statistically significant.

## Results

This study examined CP and non-CP cohorts comprising 15,848 and 62,158 patients, respectively (Table 1). The two cohorts were well matched for age, sex, and comorbidities. Patients in the CP and non-CP cohorts had mean ages of 48.5 ± 15.3 and 48.6 ± 15.4 years, respectively. Most patients were younger than 49 years (63.5%) and men (81.9%). In the study cohorts, the most common comorbidities, sorted according to the frequency of occurrence, were alcohol-related illness (45.1%), diabetes (32.2%), hypertension (25.6%), hyperlipidemia (24.1%), and CAD (9.33%).

**Table 1:** Demographic characteristics of and comorbidities in patients with and without chronic pancreatitis.

	Chronic pancreatitis		
	No	Yes	
Variable	N = 62158	N = 15848	*p*-value
**Age, year**			0.42
≤ 49	39085(62.9)	10055(63.5)	
50–64	12344(19.9)	3012(19.6)	
65+	10729(17.3)	2691(17.0)	
Mean±SD^†^	48.6(15.4)	48.5(15.3)	0.24
**Sex**			0.61
Female	11347(18.3)	2865(18.1)	
Male	50811(81.7)	12983(81.9)	
**Occupation**			<0.001
White collar	28644(46.1)	6681(42.2)	
Blue collar	24672(39.7)	6601(41.7)	
Others^‡^	8842(14.2)	2566(16.2)	
**Urbanization level**^&^			<0.001
1 (highest)	14092(22.7)	3276(20.7)	
2	18458(29.7)	4804(30.3)	
3	10560(17.0)	2630(16.6)	
4(lowest)	19048(30.6)	5138(32.4)	
**Comorbidity**			
Hyperlipidemia	14315(23.0)	3819(24.1)	0.005
Diabetes	19404(31.2)	5110(32.2)	0.01
Obesity	70(0.11)	24(0.15)	0.21
Hypertension	15684(25.2)	4049(25.6)	0.41
COPD	3398(5.47)	896(5.65)	0.36
Alcohol-related illness	27392(44.1)	7144(45.1)	0.02
Stroke	3956(6.36)	1019(6.43)	0.76
CAD	5701(9.17)	1479(9.33)	0.53
Inflammatory bowel disease	165(0.27)	70(0.44)	0.0003
Hyperparathyroidism	19(0.03)	16(0.10)	<0.001
End stage renal disease	595(0.96)	246(1.55)	<0.001

Chi-squared test;

^†^: Student’s T-test

^&^: The urbanization level was categorized by the population density of the residential area into 4 levels, with level 1 as the most urbanized and level 4 as the least urbanized.

^‡^Other occupation categories included primarily retired, unemployed, or low-income populations.

**Fig 1:**
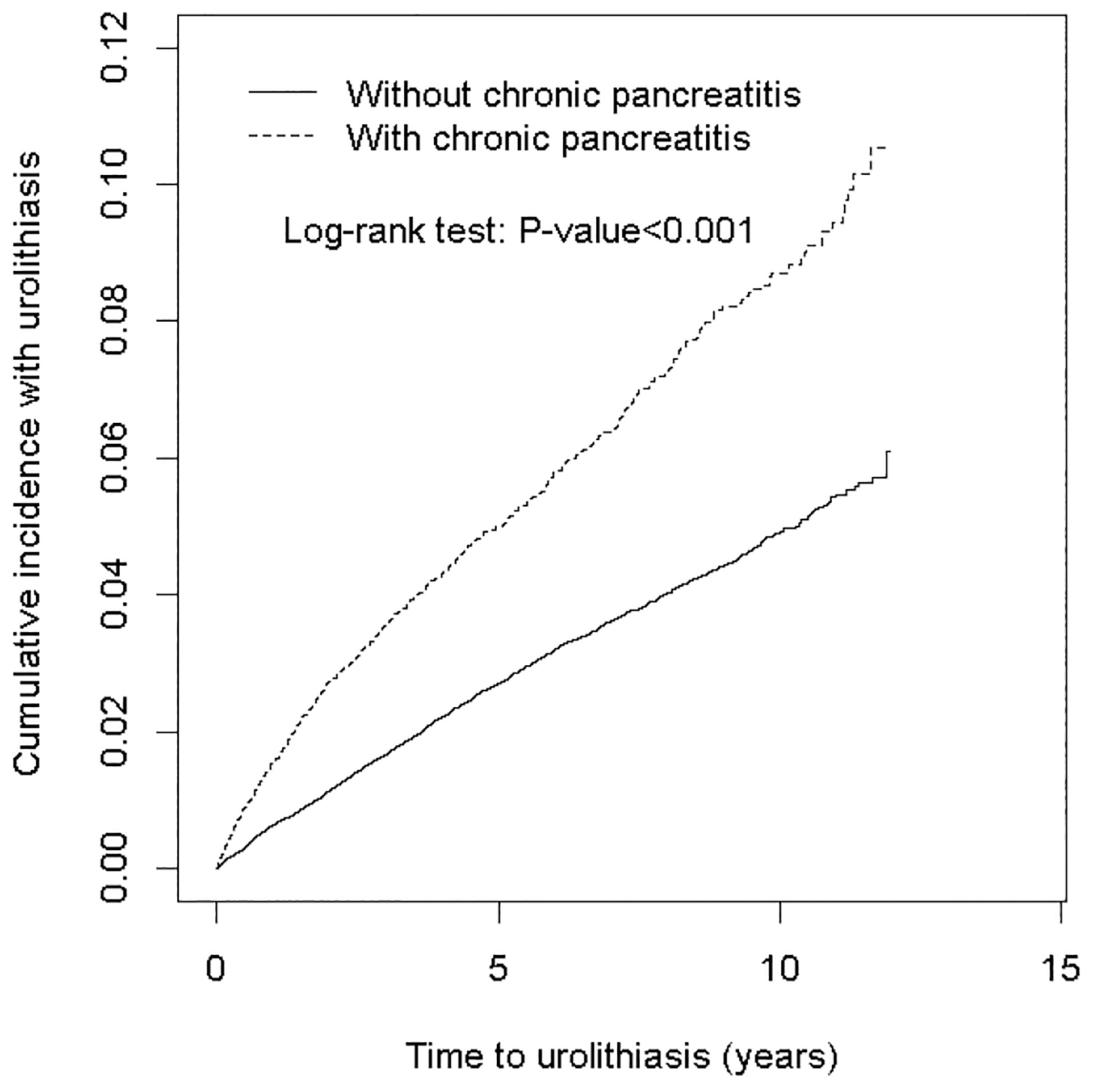
Comparison of the cumulative incidence of urolithiasis in patients with (dashed line) and without (solid line) chronic pancreatitis.

Fig 1 shows a higher cumulative incidence of urolithiasis in the CP cohort than in the non-CP cohort (log-rank test, P < 0.001). The average follow-up durations were 4.72 ± 3.28 and 5.45 ± 3.24 years for the CP and non-CP cohorts, respectively.

Table 2 shows the incidence of and risk factors for urolithiasis. The overall incidence density rates of urolithiasis for the CP and non-CP cohorts were 10.3 and 5.27 per 1,000 person-years, respectively. Compared with patients without pancreatitis, those with CP were associated with an increased risk of urolithiasis (adjusted HR [aHR] = 1.89, 95% CI = 1.74–2.06) after adjustment for age, sex, occupation, urbanization level, hyperlipidemia, diabetes, hypertension, COPD, alcohol-related illness, stroke, CAD, and end stage renal disease. The risk of urolithiasis was 1.39-fold higher in men (95% CI = 1.23–1.57) than in women. Compared with patients living in areas with level 1 urbanization, the risk of urolithiasis was 1.19-fold higher in those living in areas with level 2 urbanization (95% CI = 1.06–1.34), 1.32-fold higher in those living in areas with level 3 urbanization (95% CI = 1.15–1.50), and 1.41-fold higher in those living in areas with level 4 urbanization (95% CI = 1.25–1.58). Multivariable analysis identified hyperlipidemia (aHR = 1.26, 95% CI = 1.15–1.37), diabetes (aHR = 1.12, 95% CI = 1.02–1.22), hypertension (aHR = 1.21, 95% CI = 1.10–1.34), COPD (aHR = 1.40, 95% CI = 1.18–1.65), alcohol-related illness (aHR = 1.28, 95% CI = 1.17–1.39), and stroke (aHR = 1.23, 95% CI = 1.04–1.46) as the independent risk factors for urolithiasis. The incidence of urolithiasis was greater in the younger individuals (age 20–49: 6.32/1,000 person-years, age 50–64: 6.13/1,000 person-years, age 65+: 5.58/1,000 person-years), but there was no statistical significance.

**Table 2:** Incidence of and risk factors for urolithiasis.

Variable	Event	PY	Rate^#^	Crude HR(95% CI)	p-value	Adjusted HR^&^ (95% CI)	p-value
**Chronic pancreatitis**							
No	1786	338894	5.27	1.00		1.00	
Yes	767	74787	10.3	1.93(1.77, 2.10)	<0.001	1.89(1.74, 2.06)	<0.001
**Age, year**							
≤ 49	1720	272181	6.32	1.16(1.03, 1.30)	0.015	1.04(0.90, 1.20)	0.59
50–64	490	79976	6.13	1.11(0.97, 1.28)	0.13	1.05(0.91, 1.22)	0.48
65+	343	61524	5.58	1.00		1.00	
**Sex**							
Female	334	77086	4.33	1.00		1.00	
Male	2219	336595	6.59	1.52(1.35, 1.70)	<0.001	1.39(1.23, 1.57)	<0.001
**Occupation**							
White collar	1073	189997	5.65	1.00		1.00	
Blue collar	1113	166136	6.70	1.19(1.09, 1.29)	<0.001	1.09(1.00, 1.19)	0.05
Others^‡^	367	57548	6.38	1.12(1.00, 1.27)	0.05	1.01(0.90, 1.14)	0.83
**Urbanization level**^†^							
1 (highest)	440	93703	4.70	1.00		1.00	
2	743	125300	5.93	1.26(1.12, 1.42)	0.001	1.19(1.06, 1.34)	0.004
3	452	69396	6.51	1.38(1.21, 1.58)	<0.001	1.32(1.15, 1.50)	<0.001
4(lowest)	918	125283	7.33	1.55(1.39, 1.74)	<0.001	1.41(1.25, 1.58)	<0.001
**Comorbidity**							
**Hyperlipidemia**							
No	1791	316821	5.65	1.00		1.00	
Yes	762	96860	7.87	1.39(1.28, 1.52)	<0.001	1.26(1.15, 1.37)	<0.001
**Diabetes**							
No	1702	292545	5.82	1.00		1.00	
Yes	851	121137	7.03	1.20(1.10, 1.30)	<0.001	1.12(1.02, 1.22)	0.01
**Obesity**							
No	2548	413305	6.16	1.00		1.00	
Yes	5	376	13.3	2.08(0.86, 4.99)	0.10	-	
**Hypertension**							
No	1884	322850	5.84	1.00		1.00	
Yes	669	90831	7.37	1.24(1.13, 1.35)	<0.001	1.21(1.10, 1.34)	<0.001
**COPD**							
No	2388	395650	6.04	1.00		1.00	
Yes	165	18032	9.15	1.48(1.26, 1.73)	<0.001	1.40(1.18, 1.65)	<0.001
**Alcohol-related illness**							
No	1240	238952	5.19	1.00		1.00	
Yes	1313	174729	8.51	1.44(1.33, 1.55)	<0.001	1.28(1.17, 1.39)	<0.001
**Stroke**							
No	2386	393031	6.07	1.00		1.00	
Yes	167	20650	8.09	1.30(1.11, 1.52)	0.001	1.23(1.04, 1.46)	0.01
**CAD**							
No	2312	380246	6.08	1.00		1.00	
Yes	241	33435	7.21	1.17(1.02, 1.34)	0.02	1.06(0.92, 1.23)	0.50
**Inflammatory bowel disease**							
No	2545	412495	6.17	1.00		1.00	
Yes	8	1187	6.74	1.09(0.54, 2.17)	0.82	-	
**Hyperparathyroidism**							
No	2552	413536	6.17	1.00		1.00	
Yes	1	145	6.89	1.10(0.16, 7.77)	0.93	-	
End stage renal disease							
No	2552	410381	6.22	1.00		1.00	
Yes	1	3301	0.30	0.05(0.01, 0.34)	0.002	0.05(0.01, 0.32)	0.002

Rate^#^, incidence rate, per 1,000 person-years; Crude HR, relative hazard ratio; Adjusted HR^&^: multivariable analysis, including age, sex, occupation, urbanization level, hyperlipidemia, diabetes, hypertension, COPD, alcohol-related illness, stroke, CAD, and end stage renal disease;

^†^: The urbanization level was categorized by the population density of the residential area into 4 levels, with level 1 as the most urbanized and level 4 as the least urbanized.

^‡^Other occupation categories included primarily retired, unemployed, or low-income populations.

Table 3 shows a comparison of the incidence densities of urolithiasis in patients with and without CP according to their demographic characteristics and comorbidities. CP was associated with the development of urolithiasis in each age group (≤49 years: aHR = 2.00, 95% CI = 1.81–2.22; 50–64 years: aHR = 1.71, 95% CI = 1.40–2.09; ≥65 years: aHR = 1.54, 95% CI = 1.20–1.98) and each sex (women: aHR = 2.10, 95% CI = 1.67–2.66; men; aHR = 1.86, 95% CI = 1.70–2.04). Among the patients without comorbidities, the rate of urolithiasis increased from 2.93/1,000 person-years in non-CP patients to 8.28/1,000 person-years in CP patients. Among the patients with comorbidities, the rate of urolithiasis increased from 6.12/1,000 person-years in non-CP patients to 10.9/1,000 person-years in CP patients. Notably, the contribution of CP to the relative risk of urolithiasis was greater in patients without comorbidities (aHR = 2.81, 95% CI = 2.30–3.44) than in those with comorbidities (aHR = 1.76, 95% CI = 1.61–1.94). Moreover, CP was also associated with the subsequent development of urolithiasis in considering each occupation and urbanization level.

**Table 3:** Comparison of the incidence of urolithiasis stratified by age, sex, occupation, urbanization level, and comorbidities and Cox model-measured hazard ratio in patients with and without chronic pancreatitis.

	Chronic pancreatitis									
	No			Yes						
Variables	Event	PY	Rate^#^	Event	PY	Rate^#^	Crude HR(95% CI)	p-value	Adjusted HR^&^ (95% CI)	p-value
**Age, years**										
≤ 49	1164	221349	5.26	556	50832	10.9	2.06(1.86, 2.28)	<0.001	2.00(1.81, 2.22)	<0.001
50–64	359	66096	5.43	131	13880	9.44	1.72(1.41, 2.10)	<0.001	1.71(1.40, 2.09)	<0.001
65+	263	51449	5.11	80	10075	7.94	1.54(1.20, 1.98)	<0.001	1.54(1.20, 1.98)	<0.001
**Sex**										
Female	232	63662	3.64	102	13424	7.60	2.08(1.65, 2.62)	<0.001	2.10(1.67, 2.66)	<0.001
Male	1554	275232	5.65	665	61363	10.8	1.90(1.73, 2.08)	<0.001	1.86(1.70, 2.04)	<0.001
**Occupation**										
White collar	773	158100	4.89	300	31897	9.41	1.90(1.66, 2.17)	<0.001	1.83(1.60, 2.09)	<0.001
Blue collar	766	134896	5.68	347	31240	11.1	1.94(1.71, 2.20)	<0.001	1.93(1.70, 2.19)	<0.001
Others^‡^	247	45897	5.38	120	11651	10.3	1.90(1.53, 2.36)	<0.001	1.93(1.55, 2.40)	<0.001
**Urbanization level**^†^										
1 (highest)	330	78184	4.22	110	15519	7.09	1.66(1.34, 2.06)	<0.001	1.61(1.30, 2.00)	<0.001
2	505	102359	4.93	238	22941	10.4	2.08(1.79, 2.43)	<0.001	2.03(1.74, 2.37)	<0.001
3	318	56635	5.61	134	12761	10.5	1.86(1.52, 2.28)	<0.001	1.84(1.50, 2.25)	<0.001
4(lowest)	633	101716	6.22	285	23567	12.1	1.92(1.67, 2.21)	<0.001	1.94(1.69, 2.23)	<0.001
**Comorbidity**^**§**^										
No	263	89904	2.93	153	18486	8.28	2.81(2.30, 3.43)	<0.001	2.81(2.30, 3.44)	<0.001
Yes	1523	248990	6.12	614	56301	10.9	1.77(1.61, 1.94)	<0.001	1.76(1.61, 1.94)	<0.001

Rate^#^, incidence rate, per 1,000 person-years; Crude HR, relative hazard ratio; Adjusted HR^&^: multivariable analysis, including age, sex, occupation, urbanization level, hyperlipidemia, diabetes, hypertension, COPD, alcohol-related illness, stroke, CAD, and end stage renal disease

^†^: The urbanization level was categorized by the population density of the residential area into 4 levels, with level 1 as the most urbanized and level 4 as the least urbanized.

^‡^Other occupation categories included primarily retired, unemployed, or low-income populations.

Comorbidity^**§**^: Patients having any one of the comorbidities (hyperlipidemia, diabetes, obesity, hypertension, COPD, alcohol-related illness, stroke, CAD, inflammatory bowel disease, hyperparathyroidism, and end stage renal disease) were classified as the comorbidity group.

## Discussion

In this study, the prevalence of CP was higher in men (81.9%) and in patients younger than 49 years (63.5%; mean age: 48.5 ± 15.3 years). Additionally, alcohol-related illness was the most common comorbidity in patients with CP. These findings reflect the possibility of alcohol consumption being the main cause of CP in our cohort, although data on lifestyle and dietary habits were unavailable in our database. In addition to alcohol-related illness, our findings are consistent with those of previous studies reporting an association of CP with diabetes, hypertension, hyperlipidemia, and CAD (Table 1) [30, 31]. Although no studies have investigated the possible mechanisms for the association between CP and comorbidities, we believe the possible reasons might be related to the etiologies of CP, such as alcohol consumption, and the sequelae of CP, such as diabetes and atherosclerosis.

Our findings are consistent with those of previous studies identifying the male sex, lower urbanization level, hyperlipidemia, diabetes, hypertension, COPD, alcohol-related illness, and stroke as the independent risk factors for urolithiasis in multivariable analysis (Table 2) [32, 33]. Women are less predisposed to urolithiasis because of the inhibition of stone formation owing to higher urinary citrate levels and decreased urinary supersaturation by estrogen [34]. No definite pathophysiological mechanism exists for the association between a lower urbanization level and urolithiasis; however, it may be related to heat exposure and water supply [35]. The association between metabolic disorders and urolithiasis has been well demonstrated in the literature; the possible explanations may include decreased urine acidification due to diminished ammonium production and increased inflammation, vascular reactivity, and impaired fibrinolysis due to increased circulation glucose resulting from increased free fatty acid release [32, 33, 36]. The close association between hypertension and urolithiasis is supposedly explained by increased urinary calcium excretion because of increased blood pressure, which has been hypothesized to a result of “renal calcium leak” and “central blood volume” [37, 38]. There may be a bidirectional association between hypertension and urolithiasis since urolithiasis increases the subsequent risk of hypertension and hypertension can acidify urine by reducing urinary citrate excretion even sometimes the calcium excretion was comparable among stone formers with or without hypertension [39]. Most COPD cases are reportedly related to smoking, which may promote urinary crystallization by increasing antidiuretic hormone secretion to reduce urine output and cause renal injury by increasing oxidative stress [40]. The association between alcohol and urolithiasis remains debated; nevertheless, alcohol may enhance the risk of urolithiasis by increasing uric acid production and reducing calcium reabsorption in renal tubules [41, 42]. Moreover, urolithiasis was postulated to be related to cardiovascular disease in a meta-analysis, and this may be explained by the associated metabolic syndrome and increased urinary excretion of oxalate and urate [43]. Urolithiasis and CAD share a common pathogenesis as the process of renal calcification is similar to atherosclerotic calcification because the formation of subepithelial calcium phosphate deposits in renal papillary vessels provides the nidus for kidney stone formation and osteopontin, an important component in the urinary calcium stone matrix, is also an important factor for the development of atherosclerosis and CAD [44].

Urolithiasis has been well known for increasing the risk of chronic kidney injury or end stage renal disease due to their shared metabolic derangement and direct kidney injury by urinary infection or obstruction [45]. However, the inverse association between end stage renal disease and the risk of urolithiasis in our study may be due to the exclusion of patients with urolithiasis before the enrollment and the association between low glomerular filtration rate and decreased urinary calcium excretion [46]. Consistent with the literature, our findings support that the incidence of urolithiasis was greater in the younger individuals even though there was no statistical significance. The increased incidence of urolithiasis in middle age may be associated with the development of metabolic syndrome, increased dietary calcium, sucrose or protein intake, and increased working time [2, 47].

Our results reveal a close association of CP with urolithiasis after adjustment for age, sex, occupation, urbanization level, hyperlipidemia, diabetes, hypertension, COPD, alcohol-related illness, stroke, CAD, and end stage renal disease in Cox proportional hazards regression models (Table 3). CP was associated with the development of urolithiasis in each age group, each sex, and each occupation and urbanization level. Moreover, the contribution of CP to the relative risk of urolithiasis was higher in patients without comorbidities. The risk of urolithiasis increased in the CP cohort as the follow-up duration after CP diagnosis increased, although the follow-up duration was shorter in this cohort (Fig 1). These findings support an increased risk of urolithiasis after CP diagnosis, although we could not ascertain the causal relationship between CP and urolithiasis in this observational study.

Although no studies have explored the association between CP and urolithiasis, we postulate the possible pathophysiological mechanisms for urolithiasis development after CP diagnosis. First, CP-induced fat malabsorption may increase intestinal oxalate absorption to promote calcium oxalate urolithiasis [18]. Similar to hyperoxaluria seen in Crohn’s disease with steatorrhea, unabsorbed bile acids and fatty acids will increase the risk of oxalate urolithiasis by inducing calcium saponification to impair the binding between free calcium and oxalate within the intestinal lumens and to increase intestinal oxalate absorption [23, 48]. Furthermore, bile acids may directly increase the colonic absorption of oxalate [24]. Second, the impaired glucose metabolism and increased free fatty acid release after CP may reduce acid acidification, increase inflammation, and promote atherosclerosis to enhance urolithiasis development [32, 33, 36]. Third, alcohol consumption may increase uric acid production to promote uric acid urolithiasis. Finally, hyperparathyroidism is a possible etiology of CP and may increase the risk of urolithiasis. However, CP remained significantly related to urolithiasis development after adjustment for alcohol-related illness and hyperparathyroidism. Moreover, the number of hyperparathyroidism cases was low in our cohort.

Our study has several advantages. First, this is the first population-based cohort study on the association between CP and urolithiasis. In addition, our longitudinal database could provide a 12-year observation period for a large cohort of 1,000,000 residents of Taiwan to demonstrate the association between CP and subsequent urolithiasis. Second, more than 99% of Taiwan residents are covered by the monopolized NHI program. Therefore, we believe that the present findings provide an association between CP and urolithiasis based on the actual situation in Taiwan.

Our study has several limitations. First, our retrospective cohort study lacks information on the living environment and lifestyle. However, we used the diagnosis of COPD and alcohol-related illness to replace the habits of smoking and alcohol drinking, respectively. Occupation was classified according to the working hours indoor or outdoor, and the association between occupation and urolithiasis was nonsignificant in our study. The urbanization levels were classified based on the population density (people/km^2^), and the association between urbanization and urolithiasis was greater at lower urbanization levels. However, the association between CP and urolithiasis remained significant, irrespective of the occupation or urbanization level (Table 3). Second, we could not validate the diagnosis by individually reviewing the medical records. However, the Taiwanese government is the only buyer of the NHI program, and all insurance claims should be statutorily audited based on the standard diagnostic criteria for medical reimbursement. Medical providers will face administrative sanction and financial penalty if the diagnostic claims do not follow the national guidelines. Third, we did not analyze the actual etiology of CP. However, CP remained associated with urolithiasis even after adjustment for most of the possible confounding factors in multivariable analysis. Furthermore, alcohol-related illness was the most common comorbidity in the CP cohort, possibly implying that alcohol was the most common etiology of CP in our study. Fourth, we could not ascertain the stone type in urolithiasis although it has been reported that CP can increase the risk of oxalate urolithiasis via enteric hyperoxaluria [49, 50]. However, disregarding this factor did not reduce the association between CP and urolithiais. Finally, we acknowledge that there might be a detection bias since more abdominal imaging studies would lead to more chances to detect urolithiasis and some of patients with urolithiasis would remain asymptomatic and undetected, particularly for the control cohort. Urolithiasis could have been underestimated in the control cohort since we recruited only patients with the first three diagnoses made for claims, which might indicate symptomatic urolithiasis.

We conclude that our population-based cohort study shows a close association between CP and urolithiasis. The contribution of CP to the relative risk of urolithiasis was even greater in patients with a lower risk of urolithiasis, such as those without other comorbidities. The risk of urolithiasis increased with the duration of follow-up after a diagnosis of CP. Our findings warrant a survey and education on urolithiasis for patients with CP.

## Supporting information

S1 Table STROBE checklist-Chen.doc. STROBE statement—Checklist of items that should be included in reports of observational studies.
(DOC)

## Abbreviations

aHR: adjusted hazard ratio
CAD: coronary artery disease
CI: confidence interval
COPD: chronic obstructive pulmonary disease
CP: chronic pancreatitis
HRs: hazard ratios
ICD-9-CM: International Classification of Diseases, Ninth Edition, Clinical Modification
NHI: National Health Insurance
NHIRD: National Health Insurance Research Database
NHRD: National Health Insurance Research Database
NHRI: National Health Research Institute
SDs: standard deviations
